# PREDICTORS OF MAXIMUM STEP LENGTH IN OLDER ADULTS: ROLES FOR STRENGTH AND ACE GENOTYPE

**DOI:** 10.1093/geroni/igad104.2281

**Published:** 2023-12-21

**Authors:** Allon Goldberg, Hayden Norris, Johnathon Garber, Summer Burkhardt, Natalie Little, Joseph Sucic

**Affiliations:** College of Health Sciences, University of Michigan-Flint, Flint, Michigan, United States; University of Michigan-Flint, Flint, Michigan, United States; Michigan State University, East Lansing, Michigan, United States; University of Michigan-Flint, Flint, Michigan, United States; University of Michigan-Flint, Flint, Michigan, United States; University of Michigan--Flint, Flint, Michigan, United States

## Abstract

Deficits in step length, assessed clinically by the maximum step length (MSL) test, are associated with poor balance and falls, in older adults. Determining predictors of MSL would facilitate targeted interventions to improve balance and reduce falls in older adults. Angiotensin-converting enzyme (ACE) insertion/deletion polymorphism genotypes interact with strength to predict stepping speed, but the interplay between ACE genotype, strength and MSL is unclear. Our objective was to determine whether strength and ACE DD genotype, through main effects or interactively, are associated with MSL, in older adults. Adults (N=103, female=58.2%, mean age 72.1 years, range 65-85 years) provided demographic, health and balance confidence information, completed grip strength and MSL testing, and provided saliva for genotyping. Multiple regression determined variance in MSL explained by strength, DD genotype (present/absent), and strength x genotype interaction, while controlling for covariates (age, sex, self-rated health and balance confidence). Covariates (model 1) explained 47.7% of MSL variance, while adding grip strength (model 2) explained additional 9.7% of variance. Adding DD genotype (model 3) explained .2%, while adding a strength x genotype interaction term (model 4) explained additional 3.4%, of MSL variance. The final model explained 61% of the variance in MSL (p<.001). DD genotype did not independently predict MSL (β=-.053; p=.421), but was interactively with strength, a significant predictor (β=.216; p=.005) of MSL. Strength was a significant predictor (β=.264; p=.003) of MSL, and as a modifiable physiological factor should be a focus of rehabilitation interventions to improve MSL and reduce falls risk in older adults.

